# Electrophysiological analysis of distal, proximal, and dual compression of the median nerve^☆☆^^☆^

**DOI:** 10.1016/j.ibneur.2025.06.010

**Published:** 2025-06-23

**Authors:** Qiongfang Zhang, Yongfeng Liu, Jinhuan Zhang, Yirong Chen, Xingxian Huang, Haibo Yu

**Affiliations:** a Electromyography Room,The fourth Clinical Medical College of Guangzhou University of Chinese Medicine, Shenzhen City, Guangdong Province, China; b Department of Acupuncture,The fourth Clinical Medical College of Guangzhou University of Chinese Medicine, Shenzhen City, Guangdong Province, China

**Keywords:** Median nerve motor branch, Carpal tunnel syndrome, Cervical spondylosis with radiculopathy, Double compression syndrome, Electroneurophsiological

## Abstract

**Objective:**

Both distal and proximal compression of the median nerve can lead to hand numbness inpatients.This study aimed to dissect the electrophysiological characteristics of median nerve motor fibers inpatients with carpal tunnel syndrome (CTS) caused by distal median nerve compression, C8 - T1 nerve root - type cervical spondylosis (CRS) resulting from proximal median nerve compression, and double - compression syndrome (DCS) with CTS combined with C8 - T1 CRS, so as to verify the double - nerve compression theory, provide evidence-based basis for clinical treatment.

**Methods:**

Retrospective analysis of 30 subjects per group (CTS, CRS, DCS, controls) from a neurophysiological database. Parameters included distal motor latency (DML1-wrist, DML2-elbow), compound muscle action potential (CMAP1, CMAP2), wrist-elbow motor conduction velocity (MCV), and F-wave latency.

**Results:**

For each indicator, both the CTS group and the DCS group showed statistically significant differences from the normal group. When comparing between the CTS group and the DCS group, there were no statistically significant differences in all data indicators. When comparing the CRS group with the normal group, there were no statistically significant differences in DML1 and DML2, but there were statistically significant differences in the shortest latency of the F wave and the motor conduction velocity (MCV).

**Conclusion:**

Double compression does not cause more severe damage to the nerve compared with simple distal compression. Distal compression of the median nerve causes more severe damage to the peripheral nerve than proximal compression. For patients with double compression syndrome, clinical treatment should prioritize the treatment of the distal compression site.

## 1 Introduction

Dual compression syndrome (DCS) refers to the presence of dual compression along the same peripheral nerve travel area (Huang and Chang, 2014). This syndrome was first proposed by Upton et al. in 1973 (Upton and McComas, 1973a). It has been found that the combination of carpal tunnel syndrome (CTS) and cervical spondylotic radiculopathy (CRS) is the most common clinical type of nerve double crush syndrome (DCS) (Lo et al., 2012). Different from one of the largest studies to date involving 305 cases of CTS with generalized electrophysiological data comparing CRS (Osterman, 1988), the present trial has a more precise experimental design based on the anatomical localization of the median nerve. The motor fibers of the median nerve that innervate the hand muscles and pass through the carpal tunnel originate from the C8-T1 roots (Jajeh et al., 2024), and enters the carpal tunnel about 3 cm above the transverse carpal tunnel, innervating the radial three-and-a-half fingers. In this study, we analyzed the electrophysiological parameters of the median nerve motor branch in cases of distal entrapment (CTS),proximal entrapment (CRS of C8 - T1 anterior root injury), combined with distal and proximal entrapment (DCS), and in normal subjects. The aim was to investigate the neurophysiologic relationship between “proximal entrapment”, “distal entrapment”, and “double entrapment” of the median nerve.

## 2 Materials and methods

### 2.1 Research subjects

A retrospective analysis was carried out on the outpatients and inpatients of The fourth Clinical Medical College of GuangzhouUniversity of Chinese Medicine from November 2023 to October 2024. Three patient groups were created, each containing 30 people: CTS (CTS Group), C8-T1 radiculopathy (CRS Group) and combined (DCS Group). and a group of normal individuals. There were 30 people in each group. All participants met the inclusion and exclusion criteria.

### 2.2 Inclusion criteria

• **C8-T1 CRS Group:**
  The patients meet the "Clinical Guidelines for the Diagnosis and Treatment of Radiculopathy Caused by Degenerative Cervical Spondylosis Based on Evidence-Based Medicine (2011)" (Bono et al., 2011). CRS patients with neck pain accompanied by radiating pain in the upper limbs, sensory abnormalities (numbness, tingling, etc.) and diagnosed with C8-T1 nerve root damage through electroneurophysiological examination are selected.
• **CTS Group:**
  Patients met the diagnostic criteria for carpal tunnel syndrome as defined in the "Evidence-Based Clinical Practice Guidelines for the Management of Carpal Tunnel Syndrome" published by the American Academy of Orthopaedic Surgeons (AAOS) and the American Society for Surgery of the Hand (ASSH) in 2024 (Shapiro et al., 2025). Diagnosis was based on clinical symptoms (e.g., hand numbness, tingling, and nocturnal worsening), physical examination findings (e.g., positive Phalen sign), and electroneurophysiological examination results.
• **DCS Group:**
  Patients met the diagnostic criteria for both C8-T1 CRS and CTS. Diagnosis was confirmed through comprehensive clinical evaluation, imaging studies, and electroneurophysiological examination.
• **Normal Control Group:**

Healthy individuals without any symptoms or signs of neurological diseases were included. Cervical radiculopathy was excluded based on imaging studies, and all electroneurophysiological examination results were within the normal range.

The case-grouping process was independently conducted by two senior clinicians. They reviewed patient data according to the unified diagnostic criteria. In cases of disagreement, a consensus was reached through discussion or consultation with a third expert to ensure the accuracy and reliability of the grouping.

### 2.3 Exclusion and rejection criteria

• Patients with pacemaker implantation.
• Patients whose multiple peripheral nerve injuries (such as those combined with diabetes) have been confirmed by electroneurophysiological examination.
• Patients with local skin breakage or edema.
• Patients whose injury of the C8-T1 nerve root has not been confirmed by neuroelectrophysiological examination.
• Repeated cases: For patients who underwent multiple electrophysiological examinations for the same condition during the study period, only data from the first electrophysiological test were included.

## 3 Research methodology

A retrospective analysis was performed on the neurological electrophysiological index medical records of 120 patients. The parameters included the median nerve motor nerve conduction latency (wrist, elbow) (Distal Motor Latency, DML), (wrist, elbow) compound muscle action potential amplitude (Compound Muscle Action Potential, CMAP), (wrist - elbow segment) conduction velocity (Motor Nerve Conduction Velocity, MCV), and the shortest latency of the median nerve F wave.

The diagnostic criteria for abnormal nerve conduction: The practice guidelines of the American Association of Electrodiagnostic Medicine, the American Academy of Neurology, and the American Academy of Physical Medicine and Rehabilitation (Electrophysiologic Diagnostic Studies of Carpal Tunnel Syndrome) were used as the reference standard (Jablecki et al., 2002).

### 3.1 Test method

(1) Electrophysiological detection methods:

In this study, a Nicolet electromyography evoked potential instrument (Natus Neurolog, Inc., USA) was used. The tests were conducted in a quiet environment at a room temperature of 25–30°C. The hand was warmed as needed to maintain a palm temperature of 32.0°C or above. The subject was placed in a supine position with the muscles of the whole body relaxed, and bilateral examinations were performed for control observation.

**① Motor nerve conduction examination:**

• Recording electrode: Placed in the center of the abductor pollicis brevis muscle, at the middle and lower 1/3 of the radial side of the line between the 1st metacarpophalangeal joint and the carpometacarpal joint, on the thenar bulge. The reference electrode was placed distal to the thumb.
• Stimulation electrode: For wrist stimulation, the cathode was 6.5 cm proximal to the recording electrode, between the flexor carpi radialis and the palmaris longus tendon (the median and radial side of the wrist),with the cathode close to the recording electrode. For elbow stimulation, it was just above the brachial artery at the cubital fossa, with the cathode closer to the distal end.

Recorded data included the distal motor latency (DML), compound muscle action potential (CMAP), and motor nerve conduction velocity (MCV) of the wrist and elbow.

**② F-wave:**

• Recording electrode: Placed in the center of the abductor pollicis brevis muscle, at the middle and lower 1/3 of the radial side of the line connecting the first metacarpophalangeal joint and the carpopharopalmetacarpal joint. The reference electrode was placed distal to the thumb.
• Stimulation electrode: For wrist stimulation, the cathode was 6.5 cm away from the proximal end of the recording electrode, between the flexor carpi radialis and the tendon of the palmaris longus muscle (the median and radial side of the wrist), with the cathode facing proximal. Ten consecutive times of supramaximal stimulation were given, and the shortest latency was recorded.

(2) Statistical methods:

From November 2023 to October 2024, electrophysiological data of eligible subjects were collected. Using a computer - generated random number table, 30 cases were randomly selected from each of the CTS, CRS, DCS, and normal control groups.and their electrophysiological data (CMAP1, CMAP2, DML1, DML2, MCV, F wave) were entered into SPSS29.0 statistical software for analysis.

For measurement data conforming to a normal distribution, a one-way analysis of variance (ANOVA) is used, and the results are expressed as mean ± standard deviation. A P value less than 0.05 indicates that the difference is statistically significant. For multiple comparisons, the Tukey HSD test is employed.

For measurement data that do not conform to a normal distribution, the Kruskal-Wallis test is adopted, and the data are expressed as the median (interquartile range). A P value less than 0.05 indicates that the difference is statistically significant. For multiple comparisons, the Mann-Whitney *U* test is used, and the Bonferroni correction method is applied.

## 4 Results

Thirty cases were randomly selected from each of CTS group, CRS group, DCS group, and the normal group in the database. The electrophysiological data of each group were input into SPSS software, and statistical analysis was performed on the median nerve motor conduction and F - wave detection values of the four groups. P < 0.05 indicates a statistically significant difference.

Among the four groups, the data for CMAP1, CMAP2, and F-wave shortest latency followed a normal distribution and were analyzed using one-way ANOVA, with results expressed as mean ± standard deviation.

Post-hoc multiple comparisons (Tukey's test) revealed:Compared to healthy controls, both the CTS and DCS groups showed significant differences in CMAP1, CMAP2, and F-wave shortest latency.Between CTS and DCS groups, no statistically significant differences were observed in CMAP1, CMAP2, and F-wave shortest latency.Between CRS and healthy controls, CMAP1 and CMAP2showed no significant differences, whereas F-wave shortest latency was significantly altered.Detailed results are presented in Table 1.

**Table 1:** Comparison of distal and proximal median CMAP amplitude and The shortest latency of F- wave between groups.

Group	N	CMAP1**(**mV**)**	CMAP2**(**mV**)**	The shortest latency of F- wave**(**ms**)**
CTS group	30	6.86 ± 2.28▲	6.62 ± 2.20▲	27.11 ± 2.05▲
CRS group	30	8.01 ± 1.63●	7.81 ± 1.57●	25.40 ± 2.12 ●☆
DCS group	30	6.42 ± 2.03●**#**	6.17 ± 1.89●**#**	27.68 ± 2.53●**#**
Normal person	30	9.21 ± 1.77▲**#**	8.82 ± 1.74▲**#**	23.56 ± 2.13▲**#**☆
F		12.359	12.313	21.093
P		< 0.001	< 0.001	< 0.001

**Note:**The same symbol for the same detection index indicates a significant difference between the two groups (p < 0.05).CTS represents carpal tunnel syndrome, CRS stands for cervical spondylotic radiculopathy with C8-T1 nerve root injury, DCS represents double compression syndrome. CMAP1 represents the amplitude of the median nerve wrist motor wave, and CMAP2 represents the amplitude of the median nerve elbow motor wave.

The data of DML1, DML2, and MCV did not follow a normal distribution.

Therefore, the Kruskal - Wallis test was employed, and the data were presented as the median (interquartile range) [M (P25, P75)], as shown in Table 2.

**Table 2:** Comparison of Median Nerve DML (Wrist 1, Elbow 2) and MCV among Four Groups of Subjects.

Group	N	DML 1**（ms）**	DML 2**（ms）**	MCV**（m/s）**
CTS group	30	4.45(3.90,5.525)▲	8.90(7.875,9.50)▲	52.00(51.00,55.00) ▲
CRS group	30	2.95(2.80,3.225)●	7.15(6.675,7.625)●	55.00(53.00,60.00)●☆
DCS group	30	4.30(3.675,4.60)● #	8.40(8.025,9.30)● #	53.00(51.00,56.00) ● #
Normal person	30	3.00(2.675,3.30)▲ #	6.70(6.10,7.30)▲ #	60.00(57.00,62.25) #☆▲
H		81.027	72.126	47.115
F		0.000	0.000	0.000

**Note:** The same symbol for the same detection index indicates a significant difference between the two groups (p < 0.05). DML1 represents the motor latency of the median nerve at the wrist, DML2 represents the motor latency of the median nerve at the elbow, and MCV represents the conduction velocity of the median nerve from the wrist to the elbow segment.

Through the multiple comparisons of the Kruskal-Wallis test, it can be known that: when compared with the normal group, there are significant differences in DML1, DML2 and MCV between the CTS group and the DCS group. When comparing between the CTS group and the DCS group, there are no statistically significant differences in the data of DML1, DML2 and MCV. When comparing the CRS group with the normal group, there are no statistically significant differences in DML1 and DML2, but there is a statistically significant difference in MCV. See Table 2.

For the data with statistically significant differences after the multiple comparisons of the Kruskal-Wallis test, the Mann-Whitney *U* test is used again. After the correction by the Bonferroni method, pairwise comparisons all show statistically significant differences. See Table 3.

**Table 3:** Pairwise comparisons of the direct motor latency (wrist 1, elbow 2) and motor conduction velocity (MCV) of the median nerve among the four groups of subjects.

	**P（DML 1）**	**P（DML 2）**	**P（MCV）**
CTS group VS Normal group	0.000	0.000	0.000
DCS group VS Normal group	0.000	0.000	0.000
CRS group VS Normal group	——	——	0.001
DCS group VS CRS group	0.000	0.002	0.000
CRS group VS CTS group	0.001	0.000	0.000

**Note:** The P values are corrected using the Bonferroni method. The corrected P values for DML1 and DML2 are approximately 0.0125, and the corrected P value for MCV is approximately 0.01.

## 5 Discussion

### 5.1 Hypothesis and questioning of the nerve double entrapment theory

Upper extremity entrapment neuropathy is the most common type of peripheral neuropathy, typically caused by the compression of peripheral nerves as they pass through narrow anatomical regions (Doughty and Bowley, 2019). In 1973, Upton et al (Upton and McComas, 1973b). discovered through clinical research that 70 % of patients with carpal tunnel syndrome had symptoms of cervicothoracic nerve root damage. Consequently, the hypothesis of peripheral nerve double crush syndrome (DCS) was proposed for the first time. This hypothesis posits that when a nerve is locally damaged, minor traumatic stimuli can cause obvious damage symptoms in the remaining parts of the nerve.

Dahlin et al. confirmed that distal nerve entrapment leads to impaired retrograde transport of the axonal flow, increasing the sensitivity of the proximal nerve to compressive stimuli. Based on this, they proposed the theory of "retrograde nerve double entrapment" (Dahlin et al., 1987). Subsequent clinical and basic research has put forward various hypotheses regarding nerve double entrapment, such as "central sensitization" (Latremoliere and Woolf, 2009), "compression - induced axonal transport impairment" (Rempel et al., 1997), "compression -induced up - or down - regulation of ion channels" (Frieboes et al., 2010), "invasion of dorsal root ganglia by immune cells after peripheral nerve injury, reducing the firing threshold of neurons" (Hu et al., 2007), "impaired microcirculation" (Keir and Rempel, 2005), and "altered neurobiomechanical movement patterns" (De-la-Llave-Rincón et al., 2009).

However, clinical studies by Kwon et al. (2006) have demonstrated that although the co-occurrence rate of cervical radiculopathy and carpal tunnel syndrome is high, in many cases, there is no anatomical correlation between the proximally compressed cervical nerve root and the distally compressed median nerve in carpal tunnel hypothesis. This finding has raised questions about the authenticity of the DCS hypothesis.

### 5.2 Significance of detecting the median nerve motor branch based on anatomical theory

#### 5.2.1 Theory of median nerve anatomy

Anatomical evidence (Jajeh et al., 2024) shows that the medial root of the medial bundle of the brachial plexus and the lateral root of the lateral tract of the brachial plexus, composed of nerve fibers from the C8 - T1 root, converge into the median nerve trunk at the axilla. It runs within the biceps brachii muscle in the upper arm, is adjacent to the brachial artery at the cubital fossa, enters the forearm through the pronatorteres, reaches the carpal tunnel between the superficial and deep flexor muscles of the forearm fingers, enters the carpal tunnel about 3 cm above the transverse stria of the wrist (Zamborsky et al., 2017), penetrates the deep surface of the palmoaponeurosis to the palm, divides into terminal branches, and innervates the radial three - and - a - half fingers. The median nerve is a mixed nerve.

#### 5.2.2 Theoretical basis of pathophysiology of median nerve motor fibers

Carpal tunnel syndrome (CTS) can lead to axonal structural distortion, demyelination, and fibrosis of the median nerve. These changes can be detected by diffusion tensor imaging (DTI) (Rojoa et al., 2021). Sunderland (1978) reported that the area of motor nerve fibers within the median nerve accounted for 33 % of the total nerve fiber area. Any CRS that causes compression and irritation of the C8 - T1 nerve root, and any CTS that leads to increased carpal tunnel pressure (Rempel et al., 1999), can cause intraneural ischemia by compressing the proximal and distal median nerve. This impairs the blood - nerve interface, resulting in nerve edema, enlargement of the distal nerve segment at the compression site, and extraneural and intraneural fibrotic changes. These changes ultimately lead to demyelination of median nerve motor fibers and axonal degeneration (Schmid et al., 2020).

### 5.3 Significance of electrophysiological detection parameters of the median nerve motor branch

Electrophysiological testing is essential for identifying proximal, distal, or dual nerve injuries (Giacomini, 2006, Dillingham, 2002). Nerve conduction measurements are mainly used to reflect peripheral neuropathy (Lehmann et al., 2020). The compound muscle action potential (CMAP) is generated by applying electrical impulses to specific parts of the nerve to stimulate motor nerve fibers, which are then collected by surface electrodes and recorded. The incubation period (DML) is the time interval between the start of electrical stimulation and the appearance of the action potential (CMAP). The motor conduction velocity (MCV), which is the conduction velocity of electrical impulses between the electrical stimulation point and the recording point (IGmura, 1984), reflects the function of axons and myelin. When damage occurs, the nerve conduction elocity slows down (Hodgkin and Huxley, 1990, Waxman, 1980). F- waves are retrograde electrical impulses that are transmitted to motor neurons and then return to the distal nerve to produce secondary excitation. They can be used to assess the motor conduction function of axons along their entire length. Any involvement of a segment in the peripheral nerve conduction pathway can alter the waveform of the F-wave (Kimura, 2013). Therefore, it is a comprehensive neural circuit test. F - waves can be used to estimate motor axon conduction velocities (Panayiotopoulos and Chroni, 1996) and assist in the diagnosis of radiculopathy (Panayiotopoulos et al., 1978, Zheng et al., 2018).

### 5.4 Conclusions of this study and its clinical significance

In this clinical study, based on clinical neuroanatomy, electrophysiological data of the median nerve motor branch common to patients with distal median nerve entrapment, proximal entrapment, and double - ended entrapment were collected. These data included the shortest latency of CMAP (wrist 1, elbow 2), DML (wrist 1, elbow 2),MCV, and F - waves, and were compared and analyzed.

When comparing the CTS group with the DCS group, there were no statistically significant differences in all data indicators. For each indicator of both the CTS group and the DCS group, there were statistically significant differences when compared with the normal group. Specifically, the CMAP (wrist 1, elbow 2) was significantly reduced, the DML (wrist 1, elbow 2) was significantly prolonged, the MCV was significantly slowed down, and the shortest latency of the F wave was significantly prolonged. When comparing the CRS group with the normal group: there were no statistically significant differences in DML1 and DML2. There was a statistically significant difference in the shortest latency of the F wave. Although its value was prolonged compared with that of the normal group, it was significantly shorter than the shortest latency of the F wave in both the CTS group and the DCS group. There was also a statistically significant difference in MCV. Although its value was slowed down, it was significantly better than the average MCV of both the CTS group and the DCS group. This indicates that distal nerve compression causes more severe damage to the peripheral nerve than proximal nerve compression.Although no statistically significant differences were observed between the DCS and CTS groups, the data showed that DML1/2 was prolonged in the CTS group compared with the DCS group, and MCV was slightly slower than that in the DCS group. This suggests that the degree of injury in the DCS group was slightly lower than that in the pure CTS group, which may be related to the fact that patients in the DCS group had more obvious hand numbness symptoms and therefore sought medical attention for electrophysiological examination earlier.

Therefore, the conclusion of this study suggests that the double crush of the median nerve does not cause more severe damage to the median nerve than simple distal nerve compression, which is consistent with the clinical research results of Lo et al (Lo et al., 2012, Rehak, 2001). When there is double crush of the median nerve, it is recommended that clinically, the distal compression site should be preferentially treated.

## Ethical publication statement

We confirm that we have read the Journal’s position on issues involved in ethical publication and affirm that this report is consistent with those guidelines.

## Declarations

### Ethics approval and consent to participate

As the data in the EMG reports were anonymized and de - identified before being accessed for this study, and the analysis did not involve any direct interaction with patients or potential risks to their privacy, ethical approval was waived in accordance with the ethical review guidelines of Ethics Committee of Shenzhen Traditional Chinese Medicine Hospital.

## Financial disclosure

The research reported in this manuscript was supported by the "Sanming Project of Medicine in Shenzhen" (Grant No. SZZYSM 202311002). The funding was specifically allocated to cover the publication fees of this paper.During the course of this research,neither the authors nor their affiliated institutions received any additional financial support directly related to this study from other organizations or individuals.

## Consent for publication

All authors have read and approved the manuscript submitted.

## Funding

10.13039/501100012151Sanming Project of Medicine in Shenzhen (SZZYSM202311002).

## CRediT authorship contribution statement

**Haibo Yu:** Writing – review & editing, Resources, Project administration, Conceptualization. **Xingxian Huang:** Resources, Data curation. **Yirong Chen:** Investigation, Formal analysis, Data curation. **Yongfeng Liu:** Investigation, Data curation. **Jinhuan Zhang:** Methodology, Formal analysis. **Qiongfang Zhang:** Writing – review & editing, Writing – original draft, Methodology, Investigation, Formal analysis, Data curation, Conceptualization.

## Declaration of Competing Interest

The authors declare no competing interests.

## Data Availability

According to the requirement from the IRB of Shenzhen Traditional Chinese Medicine Hospital, all the data cannot be public due to the concerns of patients’privacy.
